# Genetic architecture of craniofacial morphogenesis: roles of *PAX3*, *PAX7*, and *PAX9*

**DOI:** 10.3389/fcell.2026.1786426

**Published:** 2026-04-30

**Authors:** Kun Wang, Ning Li, Wanqi Liu, Yong Gang Li, Fu Ren

**Affiliations:** 1 Liaoning Province Key Laboratory for Phenomics of Human Ethnic Specificity and Critical Illness (LPKL-PHESCI), Shenyang, Liaoning, China; 2 Department of Human Anatomy, Shenyang Medical College, Shenyang, Liaoning, China; 3 Shenyang Key Laboratory for Phenomics, Shenyang, Liaoning, China; 4 Department of Biochemistry and Molecular Biology, Shenyang Medical College, Shenyang, China

**Keywords:** craniofacial morphogenesis, neural crest, gene regulation, odontogenesis, PAX3, PAX7, PAX9

## Abstract

Craniofacial morphogenesis is a highly coordinated developmental process governed by complex genetic and molecular interactions. Among these, the PAX family of transcription factors plays a pivotal role in the regulation of neural crest specification, mesenchymal differentiation, and skeletal patterning. This review highlights the distinct and overlapping roles of *PAX3*, *PAX7*, and *PAX9* in craniofacial development, focusing on their gene regulatory networks, developmental mechanisms, and clinical implications. *PAX3* and *PAX7* orchestrate neural crest and cranial mesodermal pathways, whereas *PAX9* controls odontogenesis and skeletal morphogenesis. We also discuss emerging insights from systems biology, organoid models, and comparative genomics that advance our understanding of PAX-driven facial morphogenesis and its relevance to congenital craniofacial disorders.

## 1 Introduction

Craniofacial morphogenesis is among the most intricate and tightly regulated processes in vertebrate embryogenesis (Palmieri et al., 2020), involving neural crest cell (NCCs) induction, migration, proliferation, differentiation, and tissue fusion. These events are orchestrated by transcription factors, signaling pathways, and epigenetic regulators, and disruptions can lead to congenital anomalies such as cleft lip and palate, craniosynostosis, and mandibulofacial dysostosis (Minoux and Rijli, 2010; Dixon et al., 2011; Trainor, 2010; Twigg and Wilkie, 2015). Among the key regulators, the Paired box (*PAX*) family—particularly Paired box 3 (*PAX3*), Paired box 7 (*PAX7*), and Paired box 9 (*PAX9*)—plays pivotal roles in defining cranial mesenchymal identity, specifying neural crest lineages, and directing skeletal patterning, thereby shaping craniofacial morphology. While the other PAX members mainly participate in the development of non-skull facial organs such as the kidneys (Torres et al., 1995), eyes (Onuma et al., 2002), pancreas (Sosa-Pineda et al., 1997), etc., and have a weak correlation with the formation of craniofacial morphology, this article mainly focuses on in-depth exploration of *PAX3*/*PAX7*/*PAX9*.

The embryonic development of the craniofacial region originates from the migration, proliferation and differentiation of NCCs (Zhang et al., 2016). Around the fourth week of embryonic development, NCCs detach from the dorsal side of the neural tube and undergo epithelial–mesenchymal transition (EMT) before migrating to the frontal nasal prominence, maxillary prominence, and mandibular prominence to form the facial mesenchymal core (Marquez et al., 2025). These mesenchymal cells further proliferate and differentiate, participating in the formation of the jawbone, cartilage and connective tissue, promoting the growth and fusion of facial prominences, and gradually forming structures such as the nose, lips, etc (Feng et al., 2025). Meanwhile, the palate continues to develop, while the palatal process grows from both sides towards the midline and fuses, separating the oral cavity from the nasal cavity. The development of the jaw depends on the mesenchyme derived from NCCs forming the upper and lower jaw bones through intramembranous ossification, ultimately completing the overall construction of the facial shape, jawbone morphology and oral structure (Lautenschlager et al., 2017). Among them, PAX9 regulates the proliferation of palatal mesenchymal cells and participates in the morphogenesis of the palate (Sweat et al., 2020a); PAX3 is involved in the specification and migration of embryonic muscle progenitor cells (Kahsay et al., 2022); PAX7 maintains the balance of proliferation and differentiation of myogenic precursor cells (Latroche et al., 2017); PAX3 and PAX7 interact with myogenic regulatory factors (MRFs) to regulate the formation of jaw facial skeletal muscles (Qiu et al., 2020), and the three of them jointly participate in the normal establishment of the overall morphology of the craniofacial region.

Craniofacial morphology encompasses the study of the skull, facial bones, muscles, soft tissues, and teeth, focusing on their shape, proportions, and variation (Ren et al., 2024). It is central to anatomy, evolutionary biology, anthropology, and genetics, as facial features reflect both an individual’s genetic background and the evolutionary history of humans (Lacruz et al., 2019). The craniofacial skeleton consists of the cranium, which protects the brain and supports the head; the facial bones, including the upper and lower jaws, zygomatic bones, and nasal bones, which form the facial contour; and the teeth, which influence jaw development and facial shape (Matsui and Klingensmith, 2014). Soft tissues, including muscles, fat, and skin, are crucial for facial expression and contour. Craniofacial development represents a highly complex, three-dimensional process governed by polygenic regulation (Ysunza et al., 2015), where individual loci can influence multiple craniofacial modules (e.g., midface, nasal, palate), while each module is shaped by multiple loci acting in concert (Claes et al., 2018).

Both genetic and environmental factors contribute to craniofacial traits. Heritability accounts for over 60% of craniofacial variation (Relethford and Harpending, 1994), with twin studies showing particularly high heritability for facial height, width, and projection (Djordjevic et al., 2016). Nevertheless, environmental influences also shape craniofacial morphology (Reyes-Centeno et al., 2015; Harvati and Weaver, 2006; von Cramon-Taubadel, 2014). Understanding the genetic basis of craniofacial development has broad implications, from evolutionary biology to clinical genetics, especially in diagnosing and treating craniofacial malformations.

Genetic approaches have advanced our understanding of craniofacial development. Genome-wide association studies (GWAS) identify common genetic variants linked to complex traits by analyzing single-nucleotide polymorphisms (SNPs) across large populations (Visscher et al., 2012; Hirschhorn and Daly, 2005), while whole-exome sequencing (WES) targets coding regions to detect rare, high-impact variants (Wu et al., 2019; Chen et al., 2021). Together, these approaches have revealed genes critical for craniofacial shape, with GWAS consistently identifying *PAX3* among the most significant loci in both normal and disease contexts (Claes et al., 2018; Paternoster et al., 2012; Baldwin et al., 1992). Other key genes include fibroblast growth factor receptor 2 (*FGFR2*) and EPH receptor A3 (*EPHA3*), which also contribute to craniofacial variation (Claes et al., 2018; Sha et al., 2016; White et al., 2021). *PAX3* is essential for skeletal development, NCC migration, and craniofacial tissue patterning (Liu et al., 2012; Motch Perrine et al., 2014).

In summary, Craniofacial embryogenesis is a highly coordinated process involving the integration of neural crest–derived ectomesenchyme, cranial mesoderm, and pharyngeal endoderm to form the facial skeleton, jaws, muscles, and connective tissues. Following neural crest induction and epithelial–mesenchymal transition, cranial neural crest cells migrate into the frontonasal, maxillary, and mandibular prominences, where they drive facial outgrowth, patterning, and fusion. Jaw development arises from neural crest–derived mesenchymal condensations that undergo intramembranous ossification to form the maxilla and mandible, while palatal shelves elevate and fuse to separate the oral and nasal cavities. These morphogenetic events are governed by tightly regulated gene networks, in which PAX3 controls neural crest specification and migration, PAX7 maintains cranial myogenic progenitors, and PAX9 regulates odontogenic and palatal mesenchyme patterning. Together, these lineage-specific roles ensure the coordinated formation and functional integration of craniofacial structures.

## 2 PAX: gene structure, expression, and functional domains

### 2.1 PAX3

#### 2.1.1 Gene structure and functional domains

The human *PAX3* gene is located on the long arm of chromosome 2 at the q36.1 region. The *PAX3* gene consists of 10 exons within a 100 kb region and encodes a protein with 479 amino acids (Barber et al., 1999). Due to differences in mRNA processing and splicing methods, there are variations among different species. The longest isoform of the human *PAX3* gene is *PAX3e*, while in mice, the longest homologous isoform corresponds to *Pax3c* and *Pax3d* (Parker et al., 2004). The PAX3 protein is a transcription factor with an N-terminal DNA binding domain and a C-terminal anti-activating domain (TAD). The DNA binding domain is composed of the paired domain (PD), octapeptide motif (OM), and homeodomain (HD) (Boudjadi et al., 2018).

The PD, spanning amino acids 33–160, is a highly conserved bipartite DNA-binding motif comprising two helix–turn–helix (HTH) subdomains connected by a flexible linker (Hu et al., 2021). It allows PAX3 to recognize specific consensus sequences in promoters and enhancers of neural crest–and muscle-related genes, including snail family transcriptional repressor 2 (SNAI2) (Plouhinec et al., 2014), forkhead box D3 (FOXD3) (Kubic et al., 2016), myogenic factor 5 (MYF5) (Bajard et al., 2006). PD forms a synergistic complex with the key co-factor zic family member 1 (ZIC1) for neural crest induction (Milet et al., 2013), and interacts with the SRY-box transcription factor 10 (SOX10) protein to activate target genes such as microphthalmia-associated transcription factor (MITF), coordinating the differentiation, proliferation and pigment synthesis of melanocytes (Bondurand et al., 2000).

The HD, located downstream of the PD (∼residues 215–273), functions as a secondary DNA-binding module that enhances PAX3’s affinity and selectivity for composite DNA elements (Underhill and Gros, 1997; Chi and Epstein, 2002). It also mediates protein–protein interactions with other homeobox and basic helix-loop-helix (bHLH) transcription factors, enabling PAX3 to act as a transcriptional scaffold during lineage specification. Mutations in this domain often impair nuclear localization or DNA binding, causing defects in neural crest differentiation and muscle formation (Lang and Epstein, 2003).

The OM connects PD and HD. It is composed of only 8 amino acids and can mediate homodimerization/heterodimerization, altering the binding mode of PAX3 to DNA and enhancing the selective regulation of specific target genes (Chalepakis et al., 1994).

The C-terminal region (∼residues 275–479) is proline-, serine-, and threonine-rich (PST) and serves as a transactivation domain, recruiting chromatin-modifying coactivators such as p300/cAMP-response element-binding protein-binding protein (p300/CBP) and protein arginine methyltransferase 5 (PRMT5) (Kioussi and Gruss, 1994). Despite lacking a rigid secondary structure, it is essential for transcriptional activation and chromatin remodeling. Contextually, the TAD can also mediate repression via corepressors like Groucho/transducin-like enhancer of split (TLE) (Lang and Epstein, 2003). Truncating or missense mutations in this region compromise transactivation and protein stability, causing hypomorphic or dominant-negative effects associated with congenital disorders, such as Waardenburg syndrome (WS) (Tassabehji et al., 1992; Pingault et al., 2010).

#### 2.1.2 Expression and functional roles

During early embryogenesis, PAX3 is expressed in the dorsal neural tube, NCCs, and paraxial mesoderm, where it regulates neural crest induction, migration, and myogenic specification (Tremblay et al., 1995). In craniofacial regions, PAX3 marks migratory cranial neural crest cells (CNCCs), contributing to cartilage, bone, and connective tissue formation (Dastjerdi et al., 2007).

In the neural crest lineage, PAX3 protein activates SNAI2, SOX10, and FOXD3, promoting epithelial–mesenchymal transition (EMT) and migration (Lang and Epstein, 2003; Epstein et al., 1994). In melanocyte precursors, PAX3 cooperates with SOX10 to activate MITF and ret proto-oncogene (RET), regulating pigmentation, proliferation, and apoptosis resistance (Lang et al., 2005). In myogenic lineages, PAX3 protein acts upstream of MYF5 and myoblast determination protein 1 (MYOD1), promoting somite-derived muscle progenitor specification (Relaix et al., 2005).

During craniofacial morphogenesis, PAX3 modulates WNT/β-catenin, BMP, and FGF signaling pathways, influencing neural crest migration and cranial suture formation (Wu et al., 2008; Kist et al., 2005). PAX3 persists in adult muscle satellite cells, maintaining regenerative potential (Relaix et al., 2005).

**Summary:** PAX3 acts as a master regulator of neural crest and myogenic development, integrating multiple signaling pathways via its modular PD, HD, and TAD domains, thereby controlling craniofacial morphogenesis, pigmentation, muscle differentiation, and neural crest–derived tissue patterning.

### 2.2 PAX7

The *PAX7* gene, located on chromosome 1p36.13, encodes a transcription factor of approximately 497 amino acids (Shapiro et al., 1993). PAX7 and PAX3 share structural similarities, both consisting of PD, OM, HD, and TAD, and their functions are highly overlapping during embryonic development (Relaix et al., 2006). They play repetitive and complementary roles in neural crest development, skeletal muscle formation, and stem cell maintenance (Hammond et al., 2007). However, PAX7 expression is initiated later than PAX3, commencing after neural tube closure, and PAX7 is not expressed in the most dorsal region of the neural tube plate (Zhou and Conway, 2022). In the development of limb muscles, PAX7 is not expressed during the critical period of muscle precursor cell migration at E10.5, but appears in the proximal limb muscles after E11.5 (Relaix et al., 2006). Using gene knock-in experiments, replacing *Pax3* with *Pax7* in mice resulted in the conclusion that Pax7 can substitute for the function of Pax3 in the development of the dorsal neural tube, neural crest cells, and lateral plate (Zhou and Conway, 2022). However, Pax7 is not expressed during the critical period of migration, leading to the absence of limb muscles in *Pax3*-deficient mice even when *Pax7* is present (Kuang et al., 2006). In the adult stage, PAX7 is an essential factor for the maintenance of skeletal muscle satellite cells (Zhang et al., 2022).

**Summary**: PAX7 primarily functions in stem cell maintenance and late tissue homeostasis, while PAX3 preferentially directs early development and cell migration.

### 2.3 PAX9

#### 2.3.1 Gene structure and functional domains

The human *PAX9* gene, located at chromosome 14q13.3, encodes a transcription factor containing approximately 341 amino acids. As a member of the *PAX* family, it contains PD, OP, and TAD, but lacks a canonical HD. PAX9 is critical for craniofacial development, particularly in tooth, palate, and skeletal formation (Peters et al., 1998; Wang et al., 2020).

The PD spans residues 17–136 and mediates DNA-binding to canonical paired box sequences in promoters and enhancers of target genes, such as muscle segment homeobox 1 *(MSX1*), fibroblast growth factor 8 (*FGF8*), and bone morphogenetic protein 4 (*BMP4*) (Wang et al., 2020). The PD is essential for transcriptional activation and repression, integrating signals from WNT, BMP, and FGF pathways to regulate craniofacial morphogenesis.

The OM, located immediately C-terminal to the PD, functions as a protein–protein interaction module. It mediates interactions with corepressors such as TLE and may modulate transcriptional repression in a context-dependent manner (Eberhard et al., 2000). This motif contributes to the fine-tuning of gene expression during craniofacial and dental development.

The TAD of PAX9 differs substantially from those of PAX3 and PAX7. The C-terminal region of PAX9 is an atypical and non-PST-enriched activation domain, lacking a large number of proline, serine, and threonine sites (Neubüser et al., 1995). This structure makes the transcriptional activation ability of PAX9 more specific, the interaction spectrum narrower, and it is less dependent on post-translational modifications such as phosphorylation for dynamic regulation (Farley-Barnes et al., 2020).

#### 2.3.2 Expression and functional roles

PAX9 is expressed in the developing pharyngeal arches, dental mesenchyme, and palatal shelves during embryogenesis (Peters et al., 1998). It regulates the proliferation and differentiation of cranial neural crest–derived mesenchyme, coordinating odontogenesis and palatogenesis. PAX9 directly activates MSX1 and BMP4, essential for tooth and skeletal development, and interacts with FGF signaling to regulate epithelial–mesenchymal interactions (Vega-Lopez et al., 2018).

Loss-of-function mutations in PAX9 cause autosomal dominant tooth agenesis, cleft palate, and craniofacial skeletal anomalies in humans and mice, demonstrating its essential role in craniofacial patterning (Eberhard et al., 2000). Unlike PAX3 and PAX7, PAX9 lacks a homeodomain and is primarily specialized for craniofacial mesenchymal differentiation rather than myogenic or neural crest lineage specification.

**Summary:** *PAX9* is a key transcription factor regulating craniofacial and dental development. Its PD mediates DNA binding and transcriptional control, while the OM modulates protein interactions and repression. PAX9 works in concert with MSX1, BMP4, and FGF signaling to orchestrate craniofacial morphogenesis and tooth formation.

## 3 Molecular mechanisms of PAX3 in craniofacial development

### 3.1 Neural crest induction and migration

PAX3 expression is initiated early in embryogenesis within multiple progenitor populations, including the neural crest, neural plate border, somites, and myogenic precursors, reflecting its broad role in developmental patterning (Gee et al., 2011). CNCCs is a highly migratory and multipotent subset, rely on PAX3 for proper migration, proliferation, and differentiation. CNCCs contribute extensively to frontonasal and maxillofacial bones (Goovaerts et al., 2025), making PAX3 essential for normal facial and skull morphogenesis.

Persistent Pax3 expression in NCCs leads to cleft palate and defective osteogenesis in mice, highlighting the necessity of downregulating Pax3 during neural crest differentiation (Wu et al., 2008). The neural crest arises at the neural plate border (NPB) between the neural plate and non-neural ectoderm during late gastrulation and early neurulation (Williams et al., 2022). PAX3 promotes expression of other NPB specifiers, including ZIC1, msh homeobox 1 (MSX1), and SNAI2, establishing a transcriptional hierarchy that primes cells for neural crest fate (Basch et al., 2006). Cooperation with PAX7 and transcription factor AP-2 alpha (TFAP2A) maintains progenitor identity and balances neural tube and neural crest derivatives (Giniūnaitė et al., 2020).

Following induction, NCCs undergo EMT. PAX3 regulates EMT-associated genes, activating SNAI2 and FOXD3, which repress epithelial adhesion molecules such as cadherin 1 (CDH1) (Plouhinec et al., 2014). PAX3 also modulates cadherin-6B (CDH6B) and matrix metalloproteinases (MMPs) to facilitate basement membrane breakdown and delamination (Schiffmacher et al., 2014).

During late gastrulation, BMP, FGF, and WNT signals induce transcription factors including PAX3, ZIC1, and inhibitor of DNA binding 3 (ID3), establishing NPB identity. PAX3 binds PAX3-binding elements (PBEs) via its PD and HD, activating targets such as *SNAI2*, twist family bHLH transcription factor 1 (TWIST1), cellular myelocytomatosis oncogene (MYC), FOXD3, and SOX10, thereby regulating NCCs EMT, migration, and differentiation (Plouhinec et al., 2014; Kotov et al., 2024). EMT involves dynamic cadherin modulation, adherens junction dissolution, and transformation of neuroepithelial cells into migratory NCCs (Kulesa et al., 2010).

Post-EMT, PAX3 influences migration by regulating genes for motility, survival, and pathfinding, including *RET*, MET proto-oncogene (*MET*), and C-X-C chemokine receptor type 4 (CXCR4), which mediate responses to hepatocyte growth factor (HGF) and stromal cell-derived factor 1 (SDF1) (Lang et al., 2005; Xu et al., 2018). It interacts with co-factors like SOX10 and MITF to specify sublineages, such as melanocytes and peripheral neurons (Thomas and Erickson, 2008), and its loss leads to aberrant NCCs migration, characteristic of WS and other neurocristopathies (Pingault et al., 2010).

PAX3 also regulates the MITF–melanin synthesis pathway via a biphasic mechanism controlling melanocyte proliferation, migration, differentiation, and apoptosis resistance. Together with SOX10, PAX3 activates MITF (Bondurand et al., 2000) and RET (Lang and Epstein, 2003). MITF drives the expression of tyrosinase-related protein 1 (TYRP1) and dopachrome tautomerase (DCT), enzymes that catalyze tyrosine oxidation to generate eumelanin or pheomelanin (Yang et al., 2020). PAX3 represses DCT transcription to prevent premature differentiation (Kubic et al., 2008), while WNT/β-catenin signaling modulates the PAX3–SOX10–MITF balance (Kubic et al., 2008). In *Pax3* mutant mice, disruption of these pathways increases cranial neural tube defects, underscoring Pax3’s role in early cranial NCC development (Palmer et al., 2021). Experimental evidence in *Xenopus* shows pax3 and zic1 independently and synergistically regulate NCC differentiation (Sato et al., 2005).

### 3.2 PAX3 and cranial suture development

PAX3 contributes to cranial suture morphogenesis. Neonatal cranial bones remain incompletely ossified, leaving fibrous gaps between bones. Suture closure requires coordinated migration, proliferation, and differentiation of neural crest–derived mesenchyme, regulated by PAX3 (Wu et al., 2008). CNCCs migrate from the dorsal neural tube, differentiate into cartilage and bone, and influence suture closure. WNT/β-catenin, FGF, and TGFβ/BMP pathways modulate neural crest proliferation and ossification, and PAX3 dysregulation disrupts these processes, causing craniofacial asymmetry and bone defects (Ishii et al., 2015).

Failure to downregulate PAX3 during neural crest differentiation results in cleft palate and delayed ossification, partly mediated by upregulation of sclerostin domain-containing protein 1 (SOSTDC1), a BMP signaling pathway antagonist (Wu et al., 2008). Persistent expression interferes with osteogenic differentiation, contributing to craniofacial malformations.

### 3.3 Genetic disorders and functional implications

Mutations in *PAX3* cause congenital disorders, notably WS, an autosomal dominant disorder with four subtypes: WS1–WS4 (Pingault et al., 2010). WS1 features hearing loss, pigmentary anomalies, and dystopia canthorum; WS2 shares pigmentary and auditory defects without dystopia; WS3 includes limb muscle hypoplasia; WS4 involves congenital megacolon (Huang et al., 2022). In 78% of WS1/WS3 families, *PAX3* mutations were identified (Tassabehji et al., 1995), including missense, deletions, nonsense, splice-site, and insertions (Jalilian et al., 2015; Somashekar et al., 2020). Mutations typically affect the PD and HD, impairing DNA binding and transcriptional activation.

Hypomorphic *PAX3* alleles (∼20% expression) allow partial compensation by *PAX7*; double mutants display severe craniofacial, neural tube, and cardiac defects (Zhou and Conway, 2022). PAX3 interacts with WNT/β-catenin signaling; altered β-catenin function modifies severity of cranial crest–derived anomalies (Palmer et al., 2021).

### 3.4 Epigenetic regulation and environmental interactions

Aberrant methylation of PAX3 intron 4 and gene body regions is associated with neural tube defects in fetuses exposed to maternal hyperglycemia or polycyclic aromatic hydrocarbons, suggesting gene–environment interactions (Denecker et al., 2014).

### 3.5 PAX3 in cancer

In alveolar rhabdomyosarcoma (ARMS), the *PAX3–FOXO1* fusion gene drives proliferation via deregulated transcriptional programs (Linardic, 2008; Marshall and Grosveld, 2012; Crose et al., 2014). The fusion retains PAX3 DNA-binding domains fused to FOXO1’s transactivation domain, activating growth-promoting genes MYCN proto-oncogene, bHLH transcription factor (MYCN), MET, fibroblast growth factor receptor 4 (FGFR4), BCL2 apoptosis regulator (BCL2) and disrupting differentiation. PAX3 is also aberrantly expressed in melanomas, regulating MITF, SOX10, and DCT, maintaining stemness and proliferation (Moore et al., 2025).

### 3.6 GWAS evidence linking *PAX3* to craniofacial traits

**FIGURE 1:**
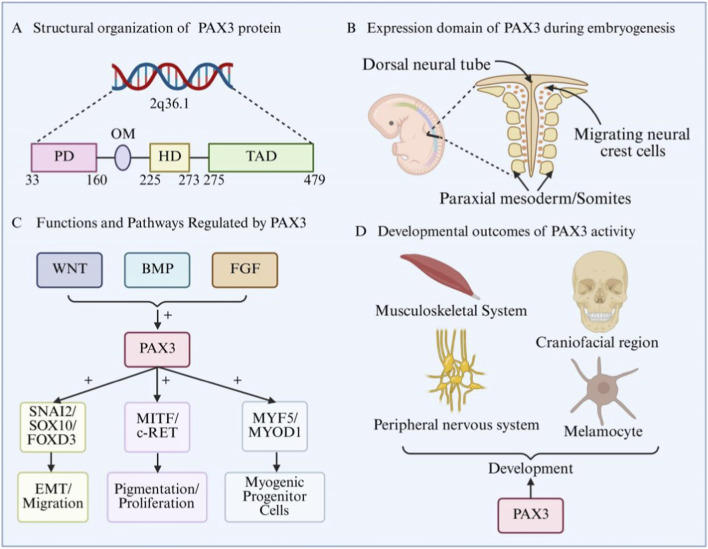
Domain architecture, expression, and developmental functions of PAX3. **(A)** Structural organization of PAX3 protein. PD: paired domain, OM: octapeptide motif, HD: homeodomain, TAD: transactivation domain. **(B)** Expression domains of PAX3 during embryogenesis. PAX3 is expressed in the dorsal neural tube and migrating neural crest cells, as well as in the paraxial mesoderm. **(C)** Regulatory interactions and developmental pathways controlled by PAX3. “+”: activation, “-”: inhibition, WNT: wnt family members, BMP: bone morphogenetic protein, FGF: fibroblast growth factor, MYF5: myogenic factor 5, MYOD1: myogenic differentiation 1, SNAI2: snail family transcriptional repressor 2, SOX10: SRY-box transcription factor 10, FOXD3: forkhead box D3, MITF: microphthalmia-associated transcription factor, c-RET: ret proto-oncogene. PAX3 acts as a transcriptional integrator downstream of the WNT, BMP and FGF signaling pathways. It activates a transcriptional cascade involving SNAI2, SOX10 and FOXD3, promoting EMT and migration, activating MITF, c-RET and MYF5, and driving melanocyte and myogenic differentiation. However, the activation relationship of WNT, BMP and FGF on PAX3 is the main effect summarized under the developmental background. At different developmental stages or cell types, the regulation of these signals on PAX3 may have dynamic changes. And c-RET itself does not directly regulate melanocytes or pigmentation, but it can indirectly affect pigment-related cells or phenotypes. **(D)** Developmental outcome of PAX3 activity. The craniofacial region, peripheral nervous system, and melanocytes are mainly derived from neural crest cells, while the musculoskeletal system is mainly derived from myogenic progenitor cells of the body segments.

GWAS studies link *PAX3* variants to nasal root position (nasion) in Europeans and other populations. SNP rs7559271 affects nasion prominence and height (Liu et al., 2012; Ueda et al., 2025). Other studies identified rs10176525 in *PAX3* associated with nasal morphology, and rs13107325 exhibits pleiotropic effects on facial traits and neurological conditions (Pickrell et al., 2016). These data highlight PAX3’s role in normal and pathological craniofacial variation (Weinberg et al., 2018). (Figure 1 shows Domain architecture, expression, and developmental functions of PAX3.)

## 4 *PAX7* in craniofacial development

### 4.1 PAX7 expression and lineage specification

PAX7 is a key regulator of craniofacial myogenesis, neural crest specification, and skeletal patterning, acting primarily within cranial mesodermal and neural crest-derived progenitors (Buckingham and Relaix, 2007; Blake and Ziman, 2014; Mayran and Drouin, 2018). Alongside its paralog PAX3, PAX7 establishes the transcriptional framework necessary for craniofacial muscle and connective tissue formation.

During early embryogenesis, PAX7 is expressed in the dorsal neural tube and cranial paraxial mesoderm, marking multipotent progenitor populations contributing to craniofacial musculature, bones, and connective tissues (Relaix et al., 2005; von Scheven et al., 2006). Loss-of-function and lineage-tracing studies demonstrate that PAX7 is essential for craniofacial muscle formation, satellite cell maintenance, and proper tissue patterning (Seale et al., 2000; Gitton et al., 2010; Borello et al., 2006).

PAX7 expression also marks neural crest–derived progenitors within the frontonasal and branchial arch regions (Buckingham et al., 2003). Spatial and temporal expression of PAX7 parallels key morphogenetic events, including neural crest migration and mesodermal condensation. In cranial mesoderm, PAX7 regulates myogenic transcription factors such as MYF5, MYOD1, and myogenin (MYOG) (Sambasivan et al., 2009), and in neural crest, it modulates SOX10 and TWIST1, promoting EMT and lineage diversification (Choi et al., 2020).

Functional ablation of *Pax7* in mouse embryos results in reduced cranial myogenic progenitors and hypoplasia of extraocular and branchial arch muscles (Relaix et al., 2006; Buckingham, 2017).

### 4.2 PAX7 in craniofacial myogenesis

PAX7 maintains, proliferates, and differentiates cranial muscle progenitors (Olguin and Olwin, 2004). It preserves progenitor identity by repressing premature differentiation through inhibition of MYOD and recruitment of chromatin modifiers that maintain enhancer quiescence (Günther et al., 2013). In postnatal tissues, PAX7 persists in satellite cells, ensuring regeneration of craniofacial muscles critical for mastication, facial expression, and eye movement (Kuang et al., 2008; Huang et al., 2013; Dumont et al., 2015). PAX7 balances symmetric and asymmetric satellite cell divisions, maintaining progenitor pools while enabling differentiation (von Maltzahn et al., 2013).

### 4.3 Neural crest–mesoderm interactions

Craniofacial morphogenesis relies on reciprocal signaling between neural crest–derived connective tissues and mesodermal muscle progenitors. PAX7 regulates molecular mediators such as FGF8, wnt family member 1 (WNT1), and BMP4 (Litsiou et al., 2005), enhancing FGF and WNT signaling to maintain mesodermal proliferation and restricting BMP to dorsal domains to guide craniofacial patterning (Thompson et al., 2004). Disruption of these feedback loops causes abnormal muscle attachment, facial asymmetry, and cranial skeletal mispatterning (Grifone and Kelly, 2007).

### 4.4 Epigenetic regulation and chromatin remodeling

PAX7 functions as a transcription factor and pioneer factor capable of remodeling chromatin in muscle progenitors (Mayran et al., 2019). It recruits coactivators such as p300/CBP to activate myogenic enhancers (Mozzetta et al., 2011) and interacts with histone methyltransferases to establish lineage-specific chromatin marks (Addicks et al., 2019). Loss of *PAX7* impairs enhancer activation and disrupts craniofacial myogenesis (Kuriki et al., 2024; Blum et al., 2012).

### 4.5 Interplay between PAX7 and PAX3

PAX7 and PAX3 share structural and functional similarity but have distinct expression domains. PAX3 is active in premigratory neural crest and early myogenic progenitors, while PAX7 maintains progenitor pools and promotes differentiation (Buckingham and Relaix, 2015; Lagha et al., 2008; Czerwinska et al., 2016). This division ensures coordinated development of craniofacial muscle and connective tissue (Harel et al., 2012; Sambasivan et al., 2011).

### 4.6 Clinical and evolutionary implications

Although no monogenic human disorder is directly linked to *PAX7* mutations, its dysregulation is associated with craniofacial muscle hypoplasia, asymmetry, and alveolar cleft repair failure (Vaivads et al., 2021). PAX7 participates in chromosomal translocations (PAX7–FOXO1) implicated in alveolar rhabdomyosarcoma (Barr, 2001). Comparative genomics suggests PAX7 arose from a PAX3 duplication, allowing subfunctionalization of cranial muscle networks, contributing to vertebrate craniofacial muscular complexity (Maczkowiak et al., 2010; Paixão-Côrtes et al., 2013).

**FIGURE 2:**
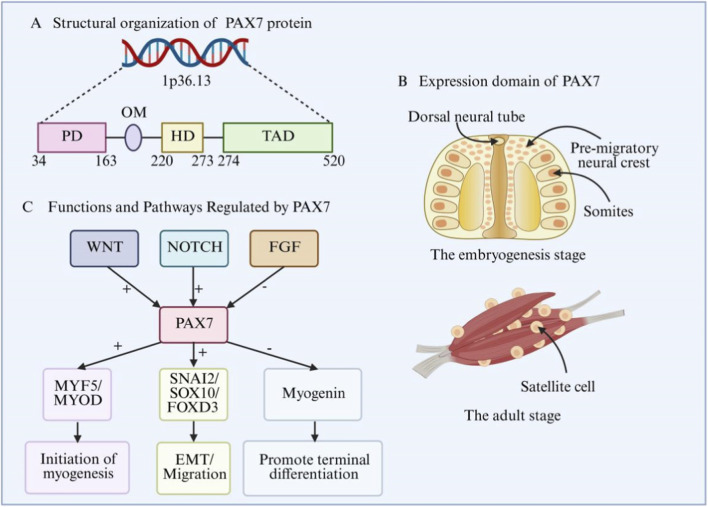
Domain architecture, expression, and developmental functions of PAX7. **(A)** Structural organization of PAX7 protein. PD: paired domain, OM: octapeptide motif, HD: homeodomain, TAD: transactivation domain. **(B)** Expression domains of PAX7 during development. During the embryonic development stage, PAX7 is mainly expressed in the dorsal neural tube, migrating pre-neural crest cells, and somites; in the adult stage, PAX7 is mainly expressed in skeletal muscle satellite cells. **(C)** Functions and signaling pathways regulated by PAX7. “+”: activation, “-”: inhibition, WNT: wnt family members, NOTCH: notch receptor, FGF: fibroblast growth factor, MYF5: myogenic factor 5, MYOD: myogenic differentiation 1, SNAI2: snail family transcriptional repressor 2, SOX10: SRY-box transcription factor 10, FOXD3: forkhead box D3, EMT: epithelial–mesenchymal transition. The expression of PAX7 is regulated by signaling pathways such as WNT, NOTCH and FGF. PAX7 activates MYF5/MYOD to initiate muscle development. It promotes EMT and cell migration by upregulating SNAI2/SOX10/FOXD3, and inhibits the myogenic factor. Thus, it plays a crucial role in embryonic development and adult muscle repair.

Summary: PAX7 integrates genetic, epigenetic, and signaling mechanisms to coordinate neural crest and mesodermal contributions to craniofacial structures. Through interaction with PAX3 and pathways such as FGF, WNT, and BMP, it ensures proper growth, patterning, and regeneration of craniofacial musculature (Figure 2 shows Domain architecture, expression, and developmental functions of PAX7).

## 5 *PAX9* in craniofacial development

### 5.1 PAX9 expression and function

PAX9 is predominantly expressed in craniofacial mesenchyme derived from neural crest cells, During craniofacial development, it is expressed in tissues contributing to jaw, palate, and teeth (Zhou et al., 2013). PAX9 is essential for odontogenesis (Das et al., 2003), particularly molar development (Shan et al., 2025). Unlike PAX3 and *PAX7*, *PAX9* does not function in neural crest induction but instead regulates later patterning and organogenesis.

### 5.2 Dental and craniofacial abnormalities

Mutations in *PAX9* frequently cause selective tooth agenesis (oligodontia), particularly permanent molars (Mendoza-Fandino et al., 2011; Jin et al., 2024; Frazier-Bowers et al., 2002). Manifestations include: Congenital absence of molars: Missing one or more permanent molars. Abnormal tooth morphology: Altered shape or size affecting occlusion. Jaw morphology changes: Severe tooth agenesis can secondarily affect mandibular and maxillary growth.

### 5.3 Tooth development pathway

PAX9 is central to odontogenesis, acting with MSX1, BMP4, and sonic hedgehog signaling molecule (SHH) to regulate tooth initiation, morphogenesis, and differentiation (Nakatomi et al., 2010). Disruption of PAX9 impairs dental lamina formation, halting bud-to-cap stage progression (Bhol et al., 2021). PAX9 ensures epithelial–mesenchymal signaling critical for molar development.

### 5.4 Craniofacial skeletal development

PAX9 expression in the pharyngeal endoderm and adjacent mesenchyme is vital for craniofacial skeletal derivatives. *PAX9* knockout mice fail to form the palatine bone, vomer, and hyoid apparatus, resulting in cleft palate and craniofacial hypoplasia (He et al., 2011). Mechanistically, PAX9 regulates mesenchymal condensation and osteogenesis via runt-related transcription factor 2 (RUNX2), SRY-box transcription factor 9 (SOX9), and BMP4 (Otto et al., 1997; Komori et al., 1997), and cooperates with paired box 1 (PAX1) for proximal cranial skeleton patterning (Wallin et al., 1996). Loss of *PAX9* disrupts epithelial–mesenchymal signaling, impairing bone and cartilage formation (Piña et al., 2024).

### 5.5 Role in odontogenesis

During the bud and cap stages, PAX9 is expressed in dental mesenchyme and interacts with MSX1 to regulate BMP4 and lymphoid enhancer binding factor 1 (LEF1), maintaining epithelial–mesenchymal signaling (Peters and Balling, 1999; Thesleff, 2003; Dassule et al., 2000; Mitsiadis et al., 2003). Disruption leads to arrested tooth morphogenesis, oligodontia, or non-syndromic tooth agenesis (Stockton et al., 2000; Nieminen, 2009; D'Souza and Klein, 2007). Most pathogenic variants affect the paired domain, reducing DNA binding and transcriptional activation (Sun et al., 2021).

### 5.6 Palatogenesis and gene regulatory networks

PAX9 regulates palatal shelf growth and fusion through proliferation and apoptosis in cranial mesenchyme. *Pax9* ^*−/−*^ mice exhibit failed palatal elevation and fusion, with disrupted TGFβ and SHH signaling (Li et al., 2019; Sasaki et al., 2007; Fu et al., 2017). PAX9 integrates into BMP, SHH, and FGF pathways, forming feedback loops that coordinate epithelial–mesenchymal interactions and craniofacial morphogenesis (Jia et al., 2020).

### 5.7 Clinical relevance

*PAX9* mutations are associated with non-syndromic tooth agenesis and cleft palate (Mitsui et al., 2014; Lee et al., 2024), primarily affecting the paired domain (Piña et al., 2023). Reduced PAX9 expression contributes to craniofacial microsomia and palatal dysplasia, highlighting its key regulatory role in skeletal and dental development (Peters et al., 1998; Farley-Barnes et al., 2020; Piña et al., 2025).

**FIGURE 3:**
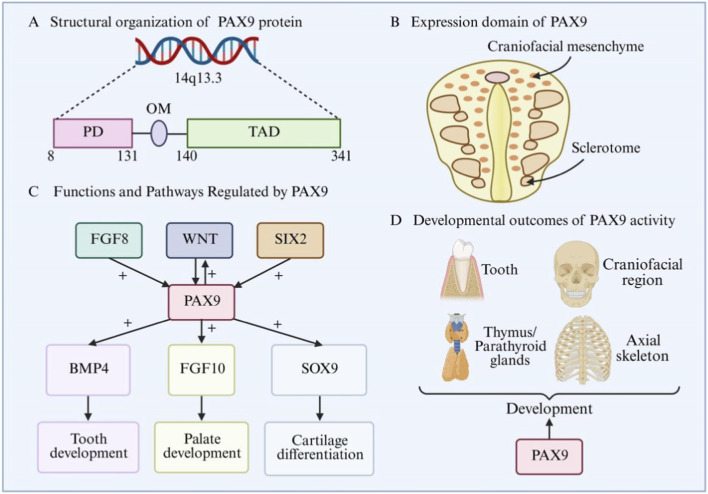
Domain architecture, expression, and developmental functions of PAX9. **(A)** Structural organization of PAX9 protein. PD: paired domain, OM: octapeptide motif, TAD: transactivation domain **(B)** Expression domains of PAX9 during embryogenesis. During embryonic development, PAX9 is mainly expressed in craniofacial mesenchyme and sclerotome. **(C)** Functions and signaling pathways regulated by PAX9. “+”: activation, FGF8: fibroblast growth factor 8, WNT: wnt family members, SIX2:SIX homeobox 2 protein, BMP4: bone morphogenetic protein 4, FGF10: fibroblast growth factor 10, SOX9: SRY-box transcription factor 9. The expression and function of PAX9 are regulated by signals such as FGF8, WNT and SIX2. It regulates tooth development by activating BMP4, palate development by FGF10, and cartilage differentiation by SOX9, playing a crucial role in cranial and skeletal development. **(D)** Developmental outcome of PAX9 activity. Normal activity of PAX9 is a necessary condition for the normal development of tissues such as teeth, cranial regions, thymus/parathyroid and axial bones. Abnormal function of PAX9 can lead to various developmental defects.

Summary: PAX9 is a central transcriptional regulator of craniofacial morphogenesis, orchestrating epithelial–mesenchymal interactions critical for skeletal patterning, odontogenesis, and palatal fusion. Its integration with MSX1, RUNX2, and SHH/BMP pathways shapes craniofacial architecture and underlies congenital malformations when disrupted.

(Figure 3 shows Domain architecture, expression, and developmental functions of PAX9.)

## 6 Integrated functional landscape of PAX3, PAX7, and PAX9 in craniofacial development

The coordinated actions of PAX3, PAX7, and PAX9 form a transcriptional framework that governs neural crest development, cranial mesoderm patterning, and craniofacial skeletal morphogenesis (Tables 1–4).

**TABLE 1:** Summary of PAX3, PAX7, and PAX9 functions in craniofacial development.

Gene	Primary expression sites	Developmental roles	Key downstream targets	Major signaling interactions
*PAX3*	Dorsal neural tube, neural crest	NCC specification, EMT, myogenesis	SOX10, MITF, TWIST1	WNT, BMP, FGF
*PAX7*	Cranial mesoderm, extraocular muscles	Myogenic progenitor maintenance	MYF5,MYOD, MEF2C	FGF, BMP
*PAX9*	Pharyngeal arches, dental mesenchyme	Skeletal and dental morphogenesis	MSX1,RUNX2, BMP4	SHH, TGF-β

WNT, wnt family members; BMP, bone morphogenetic protein; FGF, fibroblast growth factor; MYF5, myogenic factor 5; MYOD, myogenic differentiation; SOX10, SRY-box, transcription factor 10; MITF, microphthalmia-associated transcription factor; BMP4, bone morphogenetic protein 4; RUNX2, runt-related transcription factor 2; MSX1, msh homeobox; TWIST1, twist family bHLH, transcription factor 1; MEF2C, myocyte enhancer factor 2C; SHH, sonic hedgehog; TGF-β, transforming growth factor beta; EMT, epithelial–mesenchymal transition.

**TABLE 2:** Human disorders linked to PAX gene mutations.

Gene	Disorder	Mode of inheritance	Major craniofacial phenotypes	References
*PAX3*	Waardenburg syndrome types I/III	Autosomal dominant/recessive	Dystopia canthorum, nasal broadening, midfacial hypoplasia	Huang et al. (2022), Tassabehji et al. (1995), Jalilian et al. (2015), Somashekar et al. (2020)
*PAX7*	Experimental/possible congenital myogenic dysplasia	–	Facial asymmetry, cranial muscle hypoplasia	Sambasivan et al. (2009), Choi et al. (2020), Buckingham (2017), Olguin and Olwin (2004), Günther et al. (2013), Kuang et al. (2008)
*PAX9*	Non-syndromic tooth agenesis, cleft palate	Autosomal dominant	Hypodontia, jaw hypoplasia	Nieminen (2009), D'Souza and Klein (2007), Sun et al. (2021), Li et al. (2019), Sasaki et al. (2007), Fu et al. (2017), Jia et al. (2020), Mitsui et al. (2014)

**TABLE 3:** Comparative roles of PAX genes across species.

Species	PAX3 function	PAX7 function	PAX9 function	Evolutionary notes
Mouse	NCC migration and cranial patterning	Cranial myogenesis	Palatal and tooth morphogenesis	Conserved with subfunctionalization
Zebrafish	Neural crest pigment pattern	Cranial mesoderm segmentation	Pharyngeal cartilage	Gene duplication into *pax3a/pax3b*
Human	Facial morphology and variation	Muscle regeneration	Dental arch pattern	Regulatory divergence in enhancers

**TABLE 4:** Emerging experimental tools for PAX functional analysis.

Approach	Model/System	Insight provided	Example application
Single-cell RNA-seq	Mouse, human embryos	Lineage trajectories	Identify PAX3+/PAX7+ progenitor clusters
ATAC-seq/CUT&Tag	NCC and mesodermal cultures	Chromatin accessibility, enhancer usage	PAX9 enhancer binding in palatal mesenchyme
Organoid culture	hiPSC-derived facial tissues	Functional modeling	Study PAX9-regulated odontogenesis
3D bioprinting	Bioengineered cranial scaffolds	Regenerative application	PAX7-mediated myogenic repair

ATAC-seq, assay for transposase-accessible chromatin using sequencing; CUT&Tag, cleavage under targets and tagmentation.

Table 1 Highlights their complementary yet non-redundant developmental territories. PAX3 regulates NCCs induction, EMT, and early migratory competence, acting through downstream factors such as SOX10, MITF, and TWIST1, and integrating major morphogen pathways including WNT, BMP, and FGF. PAX7, by contrast, operates primarily within the cranial mesoderm, where it maintains and expands myogenic progenitors via regulatory control of MYF5, MYOD, and MEF2C. PAX9 is highly enriched in pharyngeal arch mesenchyme and dental tissues, controlling skeletal patterning and odontogenesis through transcriptional regulation of MSX1, RUNX2, and BMP4, and interacting closely with SHH and TGF-β signaling pathways. Together, these three PAX factors form a developmental axis: PAX3 → neural crest and pigment/neurogenic progenitors; PAX7 → cranial mesoderm and craniofacial muscle; PAX9 → dentoalveolar and skeletal components. This delineation mirrors their embryonic expression domains and explains the precision of their phenotypic consequences.

Table 2 Demonstrates how these distinct developmental functions translate into specific human craniofacial disorders. Mutations in *PAX3* give rise to Waardenburg syndrome types I and III, characterized by dystopia canthorum, nasal broadening, and midfacial hypoplasia, directly reflecting impaired NCCs migration and pigment specification. Although PAX7 is not definitively linked to a monogenic disorder, experimental models and isolated clinical cases suggest a role in congenital myogenic dysplasia, manifesting as facial asymmetry and cranial muscle hypoplasia. In contrast, PAX9 mutations consistently produce non-syndromic tooth agenesis, mandibular/jaw hypoplasia, and occasionally cleft palate, highlighting its central role in odontogenic epithelial–mesenchymal signaling. The gene-specific craniofacial phenotypes observed clinically underscore how disruption of individual PAX transcriptional programs yields lineage-restricted developmental abnormalities.

Table 3 Expands this perspective by examining PAX gene function across species, revealing both conservation and evolutionary divergence. In mice, Pax3, Pax7, and Pax9 retain their canonical roles in NCC migration, cranial myogenesis, and palate/tooth morphogenesis, respectively—illustrating subfunctionalization consistent with early vertebrate PAX gene duplication. Zebrafish exhibit additional partitioning: pax3a/pax3b regulate neural crest pigment patterns, pax7 controls cranial mesoderm segmentation, and pax9 orchestrates pharyngeal cartilage formation. In humans, regulatory divergence at enhancer elements likely contributes to species-specific craniofacial features such as facial shape variation, muscle regenerative potential, and dental arch patterning. These cross-species comparisons demonstrate that *PAX* genes retain deep ancestral functions while also acquiring lineage-specific regulatory refinements that shape vertebrate craniofacial diversity.

Table 4 Highlights emerging technologies that offer unprecedented resolution for dissecting PAX-mediated developmental programs. Single-cell RNA-seq enables mapping of PAX3+ and PAX7+ progenitor trajectories, revealing early lineage bifurcation within neural crest and cranial mesodermal populations. ATAC-seq and CUT&Tag provide insights into enhancer accessibility and chromatin occupancy, facilitating the identification of PAX9-bound regulatory modules in palatal and dental mesenchyme. Human iPSC-derived craniofacial organoids now serve as powerful platforms to model PAX9-dependent odontogenesis and to test gene-specific perturbations in human developmental contexts. Furthermore, 3D bioprinting technologies allow reconstruction of bioengineered cranial scaffolds and support exploration of PAX7-mediated myogenic repair, bridging developmental genetics with regenerative applications.

Together, Tables 1–4 construct a multidimensional understanding of PAX3, PAX7, and PAX9, linking their molecular architecture, developmental roles, clinical relevance, evolutionary history, and experimental interrogation. This integrated framework highlights how the PAX gene family orchestrates craniofacial morphogenesis and how emerging genomic and bioengineering tools can be leveraged to unravel their complex regulatory networks in development, disease, and regeneration.

## 7 *SOX2* interactions with PAX3, PAX7, and PAX9 in craniofacial development

### 7.1 Overview of SOX2 in craniofacial and neural crest biology

SRY-box transcription factor 2 (SOX2) is a key transcription factor that regulates the development of CNCCs. Its core function is to precisely control the sequence and quantity of the stratification, migration and differentiation of NCCs, ensuring the normal formation of cranial facial cartilage, bone, nerves, connective tissues, etc (Mandalos et al., 2014). During the early developmental stage, SOX2 is highly expressed in the neural plate and participates in maintaining cell pluripotency; when the neural crest separates from the dorsal neural tube, SOX2 expression decreases, remaining at a low level during the migration stage of NCCs (Cimadamore et al., 2011); its expression increases in the migrated neural crest-derived cells, marking the initiation of cell proliferation and differentiation. During cranial facial development, SOX2 is expressed in the neural plate boundary, anterior brain epithelium, oral epithelium and dental plate, and is located at the key interface between epithelial and neural crest-derived mesenchymal tissues (Mandalos et al., 2023). The function of SOX2 mainly serves as a chromatin-accessible precursor factor to achieve the transcriptional programs for regulating the maintenance of progenitor cells, lineage ability and epithelial-mesenchymal signal transduction (Li et al., 2023).

### 7.2 SOX2–PAX3 interactions in neural crest specification

PAX3 and SOX2 overlap in their expression at the boundary of the neural plate and jointly regulate the initiation of the neural crest. SOX2 can directly bind to the neural crest enhancer of PAX3, form a complex with caudal type homeobox 1 (CDX1), zinc finger protein of the cerebellum 2 (ZIC2), etc., and synergistically activate the transcription of PAX3 (Sanchez-Ferras et al., 2012).

When neural crest cells start to migrate, SOX2 expression decreases while PAX3 remains highly expressed; while neural tube cells retain high SOX2 expression and decreased PAX3 expression (Cimadamore et al., 2011). SOX2 directly activates N-cadherin to enhance cell adhesion and inhibit EMT; it also downregulates PAX3 and SNAI2, maintaining the stemness of the neural plate (Mandalos et al., 2014). Meanwhile, PAX3 and its homolog PAX7 can directly inhibit SOX2 expression, releasing its inhibition on the neural crest program and promoting the specialization of NPB cells into neural crest cells (Rondon et al., 2025).

SOX2 and PAX3 form a dynamic regulatory axis that maintains a balance during neural crest development: they act cooperatively to initiate the neural crest program in the early stage, and then antagonize each other to complete the fate separation between the neural plate and the neural crest in the later stage.

### 7.3 SOX2 and PAX7 in cranial progenitor maintenance

SOX2 and PAX7 jointly bind to the enhancers of target genes such as *SOX9*, *FOXD3*, and *MYC* in cranial progenitor cells, mainly the precursors of cranial neural crest, to form a stem cell regulatory complex, maintaining the self-renewal and pluripotency of cranial progenitor cells (Rondon et al., 2025; Kerosuo and Bronner, 2016). In the dorsal neural tube, SOX2 and PAX7 are co-expressed, forming a stable precursor pool, providing a cellular source for the migration of cranial neural crest (van Rijn et al., 2015).

SOX2 acts as a pioneer transcription factor, which can directly bind to compact chromatin, open local structures, allowing other transcription factors to bind, thereby maintaining cell pluripotency and preventing premature differentiation (Dodonova et al., 2020). PAX7 strengthens the cranial neural crest lineage-specific transcription program, stabilizing cell identity (Mayran et al., 2018). The two work together to stabilize the progenitor cell pool during craniofacial morphogenesis, providing a continuous cellular source for normal craniofacial development.

### 7.4 SOX2–PAX9 axis in odontogenesis and palatogenesis

Unlike PAX3 and PAX7, which act primarily in neural and mesodermal compartments, PAX9 functions in neural crest–derived mesenchyme, whereas SOX2 is prominent in craniofacial epithelium. Their interaction exemplifies epithelial–mesenchymal crosstalk.

SOX2 is mainly expressed in the dental epithelium, including the dental bud, cervical ring, and enamel knot, and it participates in regulating epithelial proliferation, differentiation, and the formation of the enamel knot signaling center (Yu et al., 2020); while PAX9 is mainly expressed in the dental mesenchyme, and it participates in regulating mesenchymal specialization and tooth formation (Chu et al., 2023). SOX2 and PAX9 form a regulatory loop through the epithelial-mesenchymal signal dialogue, and they are the key nodes for the transition from the bud stage to the cap stage of tooth development (Juuri et al., 2012).

SOX2 is highly expressed in the palatal epithelium, regulating palatal plate extension, epithelial differentiation, and palatal sulcus formation (Sweat et al., 2020b); PAX9 is widely expressed in the palatal mesenchyme, regulating palatal plate proliferation, elevation, and fusion. SOX2 and PAX9 collaborate to regulate signaling pathways such as SHH, BMP4, and FGF, driving normal growth and closure of the palatal plate (Zhou et al., 2013).

The SOX2-PAX9 axis is a crucial regulatory core for the initiation of tooth development and the morphogenesis and fusion of the palatal plate; the absence of either of their functions leads to tooth development arrest and cleft palate.

**Summary:** SOX2 functions as a pivotal upstream regulator that interfaces with PAX3, PAX7, and PAX9 across neural, mesodermal, and epithelial–mesenchymal compartments, respectively. The coordinated SOX–PAX regulatory axis ensures balanced progenitor maintenance, lineage specification, and tissue patterning during craniofacial morphogenesis, and its disruption may underlie phenotypic variability in craniofacial disorders.

## 8 Limitations and unresolved questions in PAX-Mediated craniofacial development

Although significant progress has been made in understanding the roles of PAX3, PAX7, and PAX9 in craniofacial morphogenesis, there are still some limitations. PAX proteins typically exhibit overlapping expression domains and partial redundancy in function. For instance, PAX3 and PAX7 have structural similarities and overlapping roles in neural crest and dorsal neural tube development, complicating the attribution of specific developmental outcomes to a single gene. Although downstream targets such as MSX1, BMP4, SOX10, and MYOD are frequently cited, the distinction between direct transcriptional targets and indirect regulatory effects remains unclear. Many targets are inferred from expression changes rather than being verified binding sites. Craniofacial morphogenesis depends on mutual epithelial-mesenchymal signaling, yet most studies analyze the functions of PAX genes in individual tissues. Additionally, most mechanistic insights come from mouse and zebrafish models, which may not fully encompass human craniofacial development.

The current research has largely outlined the basic framework of the role of the PAX genes, but there are still many unresolved issues. To what extent can PAX7 compensate for the loss of PAX3 in the specification of cranial neural crest? How do the mesenchymal signals regulated by PAX9 affect the behavior of epithelial stem cells? Which PAX regulatory elements are specific to humans? What is the genome-wide binding map of PAX proteins in the craniofacial tissues? How do the enhancer background and chromatin state affect the specificity of PAX binding? Is there a direct transcriptional connection between the epithelial SOX2 network and the interstitial PAX9 targets?

Overall, the current knowledge regarding the roles of PAX3, PAX7, and PAX9 in craniofacial development is limited by functional redundancy, incomplete target validation, limited cross-tissue integration, and species-specific differences. Addressing these limitations will require integrating multi-omics approaches, human-related model systems, and quantitative genotype-phenotype analyses. Solving these challenges is crucial for translating developmental insights into predictive diagnostics and regenerative therapies for craniofacial disorders.

## 9 Future directions

Despite significant advances in defining the roles of PAX3, PAX7, and PAX9 in craniofacial development, many fundamental questions remain unresolved. Future studies must bridge molecular, developmental, and translational approaches to unravel how PAX-dependent gene regulatory networks orchestrate craniofacial morphogenesis across time and tissue lineages.

First, high-resolution lineage tracing, particularly using inducible Cre recombinase/CRISPR-barcoding systems (site-specific recombination and gene-editing tools for high-precision cell fate mapping) and single-cell multiomics, will be essential to map how PAX-expressing progenitors diversify into neural crest–derived, mesoderm-derived, and odontogenic populations. Although *PAX3* and *PAX7* share overlapping expression domains, the molecular logic underlying their partial redundancy and lineage-specific specialization—especially in human craniofacial tissues—remains incompletely understood. Integrating single-cell RNA sequencing (scRNA-seq), single-cell assay for transposase-accessible chromatin using sequencing (scATAC-seq), cleavage under targets and tagmentation (CUT&Tag), and spatial transcriptomics in human and animal embryos will help clarify how chromatin state and enhancer logic drive differential PAX gene functions.

Second, the emergence of human induced pluripotent stem cell (iPSC)-derived craniofacial organoids, including neural crest, skeletal, muscle, and dental organoids, represents a major frontier for dissecting PAX-dependent developmental mechanisms. These platforms offer unprecedented opportunities to model PAX9-dependent odontogenesis, PAX7-regulated myogenesis, and PAX3-mediated NCC EMT and migration in human-relevant systems. Coupling these organoids with genome engineering—base editing, prime editing, and enhancer perturbation—will allow detailed assessment of variant pathogenicity and gene–gene interactions not feasible in animal models.

Third, comparative genomics and evolutionary developmental biology (evo-devo) provide new avenues to understand how *PAX* genes diversify craniofacial architecture across species. The existence of *pax3a*/*pax3b* paralogs in teleosts, divergence of *PAX9* enhancers in primates, and muscle-specialization roles of *PAX7* raise compelling questions about the evolutionary plasticity of PAX regulatory elements. Systematic mapping of conserved vs. species-specific enhancers will help link PAX regulatory evolution to human craniofacial diversity.

Fourth, PAX genes hold considerable promise for translational and regenerative medicine. Advances in 3D bioprinting, scaffold-based tissue engineering, and bioactive hydrogels suggest that modulating PAX7 pathways may enhance craniofacial muscle repair, while PAX9 pathway engineering may improve tooth regeneration or bioengineered dental implants. Understanding how to precisely control PAX expression and chromatin accessibility will be crucial for developing safe regenerative strategies.

Finally, given the strong association of *PAX3* mutations with WS, *PAX9* with tooth agenesis, and *PAX7* with craniofacial muscle defects, future research should prioritize integrated clinical-genomic databases that link patient genotypes, enhancer variants, and phenotypic outcomes. Such efforts will enable improved diagnostic interpretation of noncoding variants and support precision-medicine approaches in craniofacial genetics.

Collectively, emerging technologies—from multiomics and organoids to 3D bioprinting and regulatory genomics—will transform our understanding of how PAX genes shape craniofacial form and function. Continued integration of molecular biology, developmental genetics, quantitative morphometrics, and translational modeling will be essential to fully elucidate the complexity and biomedical relevance of the PAX regulatory network.

## Glossary

PAX: paired box
NCCs: neural crest cells
EMT: epithelial–mesenchymal transition
MRFs: myogenic regulatory factors
GWAS: genome-wide association studies
FGFR2: fibroblast growth factor receptor 2
EPHA3: EPH receptor A3
PD: paired domain
HD: homeodomain
OM: octapeptide motif
TAD: transactivation domain
HTH: helix–turn–helix
SNAI2: snail family transcriptional repressor 2
FOXD3: forkhead box D3
MYF5: myogenic factor 5
ZIC1: zic family member 1
SOX10: SRY-box transcription factor 10
MITF: microphthalmia-associated transcription factor
bHLH: basic helix-loop-helix
PST: proline-, serine-, and threonine-rich
p300/CBP: p300/cAMP-response element-binding protein-binding protein
PRMT5: protein arginine methyltransferase 5
WS: waardenburg syndrome
TLE: groucho/transducin-like enhancer of split
CNCCs: cranial neural crest cells
EMT: epithelial–mesenchymal transition
RET: ret proto-oncogene
MYOD1: myoblast determination protein 1
MSX1: muscle segment homeobox 1
FGF8: fibroblast growth factor 8
BMP4: bone morphogenetic protein 4
NPB: neural plate border
MSX1: msh homeobox
TFAP2A: transcription factor AP-2 alpha
CDH1: cadherin 1
CDH6B: cadherin-6B
MMPs: matrix metalloproteinases
ID3: inhibitor of DNA binding 3
PBEs: PAX3-binding elements
TWIST1: twist family bHLH transcription factor 1
MYC: cellular myelocytomatosis oncogene
MET: MET proto-oncogene
CXCR4: C-X-C chemokine receptor type 4
HGF: hepatocyte growth factor
SDF1: stromal cell-derived factor 1
DCT: dopachrome tautomerase
TYRP1: tyrosinase-related protein 1
SOSTDC1: sclerostin domain-containing protein 1
ARMS: alveolar rhabdomyosarcoma
MYCN: MYCN proto-oncogene, bHLH transcription factor
GWAS: genome-wide association studies
SNPs: single-nucleotide polymorphisms
WES: whole-exome sequencing
*MYOG*: myogenin
WNT1: wnt family member 1
SHH: sonic hedgehog signaling molecule
RUNX2: runt-related transcription factor 2
SOX9: SRY-box transcription factor 9
LEF1: lymphoid enhancer binding factor 1
SOX2: SRY-box transcription factor 2
CDX1: caudal type homeobox 1
ZIC2: zinc finger protein of the cerebellum 2
scRNA-seq: single-cell RNA sequencing
scATAC-seq: single-cell assay for transposase-accessible chromatin using sequencing
CUT&Tag: cleavage under targets and tagmentation
iPSC: induced pluripotent stem cell
evo-devo: evolutionary developmental biology
